# Modified Dhole-inspired optimization for maximum power extraction in photovoltaic systems under partial shading

**DOI:** 10.1038/s41598-026-47686-1

**Published:** 2026-04-24

**Authors:** Resat Celikel, Omur Aydogmus, Musa Yilmaz

**Affiliations:** 1 Faculty of Technology, Department of Electrical and Electronics Engineering, Firat University, Elazig, 23200 Turkey; 2 Bourns College of Engineering, Center for Environmental Research and Technology, University of California at Riverside, Riverside, CA 92521 USA; 3 Department of Electrical and Electronics Engineering, Batman University, Batman, 72100, Turkey

**Keywords:** PV system, Partial shading, MPPT, Optimization, Dhole-inspired, Energy science and technology, Engineering, Mathematics and computing

## Abstract

In photovoltaic systems, PSC occur when PV panels are exposed to nonuniform solar irradiance levels. Extracting the maximum power from PV systems operating under PSC represents a complex and challenging task for MPPT algorithms. Optimization-based MPPT techniques have therefore gained significant attention due to their ability to achieve fast convergence and high efficiency under such conditions. In this study, a novel M-DHO algorithm is proposed by integrating the DHO algorithm, which is inspired by the cooperative hunting behavior of the Asiatic wild dog, with a Levy flight strategy to enhance global search capability. Especially with Levy flight support, the M-DHO algorithm eliminates the problems of fast convergence and getting stuck in a local minimum. Furthermore, while the fast convergence problem is eliminated, the Levy Flight algorithm allows reaching the global maximum value faster with high accuracy in complex optimization problems. Nine distinct PSC scenarios are created across six different voltage regions, and the performance of the proposed M-DHO algorithm is comparatively assessed against GWO, WOA, FPA, and the conventional DHO algorithm. Simulation results demonstrate that the proposed M-DHO algorithm achieves faster convergence to the global maximum power point and higher tracking efficiency compared to the benchmark algorithms. When averaged over all scenarios, M-DHO achieved an average extracted power of 838.58W, tracking speed of 0.15s and an average tracking efficiency of 99.52%, outperforming other algorithms.

## Introduction

Renewable energy sources have gained increasing importance due to their potential to reduce environmental problems and provide alternatives to fossil fuels. Among renewable energy technologies, PV energy systems play a significant role^1^. PV panels can be connected in series and parallel to obtain the desired voltage and power levels. However, due to the relatively low efficiency of PV panels, it is essential that these systems operate continuously at their maximum efficiency^2,3^.

The maximum power obtained from PV panels varies depending on solar irradiance and temperature conditions. For this reason, MPPT algorithms have been developed to ensure continuous extraction of maximum power from PV systems^4^. Conventional MPPT algorithms widely reported in the literature include Perturb and Observe (P&O)^5,6^, Incremental Conductance (Inc)^7,8^, and Open-Circuit Voltage (0.8Voc) methods^9,10^. An effective MPPT algorithm is expected to rapidly track the maximum power point under changing atmospheric conditions while minimizing power oscillations in steady-state operation. However, in conventional MPPT methods, selecting a small duty-cycle increment improves steady-state accuracy but slows convergence speed, whereas a large increment accelerates convergence but increases steady-state power oscillations. To mitigate these drawbacks, modified versions of conventional algorithms^11–13^, as well as fuzzy logic–based^14^, artificial intelligence–based^15,16^, and optimization-based MPPT algorithms^17^, have been proposed.

PSC occur when PV modules within an array are subjected to nonuniform irradiance levels. Under PSC, multiple local maximum power points appear; however, only one corresponds to the GMPP, which must be accurately identified by the MPPT algorithm. Conventional MPPT algorithms generally fail to perform effectively under PSC. Therefore, several modified MPPT techniques have been proposed to improve performance under both PSC and uniform irradiance conditions^18–20^. In recent years, scanning-based MPPT algorithms have also become an important research topic^21^. Among these, voltage scanning techniques have emerged as effective alternatives, particularly under severe PSC conditions^22^. In a recent study based on transient voltage analysis, a tracking efficiency of 99.68% with a convergence time of 0.013 s was reported for a PV system operating under complex PSC scenarios^23^.

One of the methods used to obtain maximum power from PV systems operating under PSCs is the PV array reconfiguration technique. An approach for reconfiguring solar PV arrays under PSCs has been described; in this approach, an adaptive PV array is connected to the fixed part of a PV system through a matrix of switches. These switches reconfigure solar cell connections based on a real-time control algorithm. The disadvantage of this method is that it requires a large number of switches^24^. Another method is to generate a duty cycle using an MPPT algorithm to maximize the power of the PV system using a DC-DC converter. In this method, there is a time delay in capturing the MPP point because search mechanisms are used.

Recently, optimization-based MPPT algorithms have been extensively employed to extract maximum power from PV systems operating under PSC. Their main advantages include fast convergence to steady-state operation and reduced power oscillations under steady-state conditions. Nevertheless, achieving the highest possible tracking efficiency under PSC requires MPPT algorithms with high accuracy and rapid response. Consequently, new and improved optimization techniques continue to be developed.

PSO is one of the earliest optimization-based algorithms applied to MPPT applications. PSO is an intelligent optimization algorithm inspired by the social behavior of bird flocks and used to achieve maximum power in PV systems in PSCs. A fundamental problem with traditional PSO is its random nature. Typical PSO has limitations such as slow convergence, poor local search capabilities, more parameters to adjust, and oscillations around the MPP^25^. Initial studies primarily aimed to eliminate steady-state power oscillations and achieve maximum power extraction under both uniform irradiance and PSC conditions^26,27^. Subsequent research focused on improving the convergence speed of PSO-based MPPT algorithms^28^. GWO-based MPPT algorithms were later introduced, demonstrating higher efficiency compared to PSO^29^. The CSA gained attention due to its lower steady-state power oscillations^30^, while the WOA exhibited superior performance compared to PSO and GWO across different PV system configurations^31^.

Ongoing studies have proposed numerous optimization-based MPPT algorithms aimed at improving convergence speed and tracking efficiency, including Henry Gas Solubility Optimization^32^, Musical Chairs Algorithm^33^, Roach Infestation Optimization^34^, Ant Colony Optimization^35^, Horse Herd Optimization^36^, Arithmetic Optimization Algorithm^37^, Falcon Optimization Algorithm^38^, Teaching–Learning Optimization^39^, Dandelion Optimizer^40^, Jaya Optimization^41^, Sooty Tern Optimization^42^, Artificial Bee Colony^43^, Harris Hawk Optimization^44^, Seagull Optimization Algorithm^45^, Rat Swarm Optimizer^46^, Team-Game Optimization^47^, Coot Optimizer Algorithm^48^, and Flower Pollination Algorithm (FPA)^49^. Research in this field remains active and continues to expand.

DHO algorithm is a recently proposed optimization technique introduced by Romeh et al. in 2025, offering high convergence speed and efficiency^50^. Levy flight is a mathematical strategy widely used in search mechanisms to enhance exploration capability, and its integration with various optimization algorithms has been shown to improve overall performance^51–53^. In this study, a M-DHO method is developed by combining the DHO algorithm with the Levy flight approach and is applied as an MPPT algorithm for PV systems. The stochastic approaches given above include many methods such as swarm intelligence techniques, pheromone-based foraging techniques, memory-based strategies, evolution-based strategies, predator-prey relationship modeling approaches, social hierarchy approach, and strategies modeling probabilistic and physical laws. The common goal of these models is to prevent premature convergence, optimize the balance between exploration and exploitation, and reduce or eliminate generalizability problems in high-dimensional optimization problems. In some very recently developed algorithms, adaptive leadership mechanisms have been developed by improving these cooperative strategies. On the other hand, many new methods involve modifications of existing foraging algorithms. The parameters of newly developed algorithms are based on well-defined or known theoretical algorithms. However, the success of adaptive algorithms remains a subject of debate. The DHO algorithm, particularly when integrated with the Levy Flight mechanism, combines hierarchical leadership dynamics with cooperative hunting strategies to effectively regulate the balance between exploration and exploitation. This integration mitigates the issues of rapid convergence and entrapment in local optima, while demonstrating strong performance in approaching the global optimum. The proposed method is evaluated under PSC using a MATLAB/Simulink-based PV system model consisting of six series-connected PV arrays. The M-DHO algorithm demonstrates high efficiency across nine challenging PSC scenarios, including cases with multiple power peaks within the same voltage region, closely spaced power peaks across different voltage regions, and low-voltage global maximum power points.

The aim of this article is to optimally achieve the exploration-exploitation balance in single-objective optimization algorithms using adaptive mechanisms and to quickly find the optimal value in a complex optimization problem. For this purpose, the M-DHO algorithm is proposed. Unlike the typical dominance-based leadership seen in other swarm-organizing species, the role of the Lead Vocalizer is based on guiding and coordinating the swarm rather than establishing physical dominance. The algorithm dynamically adjusts the Lead Vocalizer’s influence, establishing an adaptive balance between global exploration and local exploitation, preventing the search from remaining diverse and converging to suboptimal solutions prematurely. While preventing rapid convergence, it enables the rapid and highly accurate attainment of the global maximum value through a systematic search method thanks to the Levy flight algorithm instead of random exploration. Furthermore, the proposed algorithm is comparatively evaluated against widely used GWO-, FPA-, and WOA-based MPPT algorithms. Simulation results are presented graphically, and a detailed performance analysis is conducted. The M-DHO–based MPPT method exhibits lower computational complexity compared to existing high-performance MPPT algorithms while maintaining stable and high tracking performance across all voltage regions. Moreover, when averaged over the nine PSC scenarios, the proposed method achieves superior tracking efficiency.

## PV system and partial shading

A PV panel is modeled using a current source, a diode, and two resistances. Considering the effects of temperature and solar irradiance, the output current of the PV cell is expressed by Eq. 1. The output current obtained from a PV module is subsequently described by Eq. 2 and Eq. 3^54,55^.

1 $$\begin{aligned} & I = I_{PV} - I_D - I_{RP} \end{aligned}$$

2 $$\begin{aligned} & I = I_{PV} - I_{0}\left[ \exp \left( \frac{V + R_{s}I}{a}\right) - 1\right] - \frac{V + R_{s}I}{R_{P}} \end{aligned}$$

3 $$\begin{aligned} & a = \frac{N_{s} n k T}{q} \end{aligned}$$

where $I_0$ refers to the reverse saturation current (leakage current) of the diode. $I_d$ is diode current, $I_{RP}$ is shunt current, $I_{PV}$ is the photovoltaic current, *I* is output current, $R_s$ is the series resistance, $R_p$ is parallel rsistance, *V* is output voltage, *a* is the ideality factor, $N_s$ is the number of series-connected cells, *n* is the diode ideality constant, *k* is the Boltzmann constant ($1.3806503\times 10^{-23}\,\mathrm {J/K}$), *T* is the cell temperature (Kelvin), and *q* is the electron charge ($1.60217646\times 10^{-19}\,\textrm{C}$). The current generated by the PV cell by the effect of light is given in Eq. 4.

4 $$\begin{aligned} I_{PV} = \left( I_{PV,n} + K_{I}(T - T_{n})\right) \frac{G}{G_{n}} \end{aligned}$$

where $I_{PV,n}$ refers to the current generated at $25\,^\circ \textrm{C}$ and $1000\,\mathrm {W/m^2}$, $T_n$ refers to the nominal temperature (Kelvin), *G* refers to the radiation value on the panel surface ($\mathrm {W/m^2}$), and $G_n$ refers to the nominal radiation value ($\mathrm {W/m^2}$). The saturation current of the diode ($I_0$) is given in Eq. 5.

5 $$\begin{aligned} I_{0} = \frac{I_{SC,n} + K_{I}(T - T_{n})}{\exp \left( \frac{V_{OC,n} + K_{V}(T - T_{n})}{a}\right) - 1} \end{aligned}$$

where $I_{SC,n}$ is the nominal short-circuit current, $V_{OC,n}$ is the nominal open-circuit voltage, $K_I$ is the current coefficient, and $K_V$ is the voltage coefficient.

In the simulation study, six KU340-8BCA panels manufactured by Kyocera Solar, with parameters given in Table 1, were connected in series. Under standard test conditions of $25\,^\circ \textrm{C}$ and $1000\,\mathrm {W/m^2}$, a PV array producing $2042\,\textrm{W}$ at an output voltage of $247.2\,\textrm{V}$ was formed.

**Table 1:** Parameters of Kyocera Solar KU340-8BCA.

Symbol	Quantity	Value	Unit
$P_{MPP}$	Max. power at MPP	340.312	W
$V_{MPP}$	Voltage at MPP	41.2	V
$I_{MPP}$	Current at MPP	8.26	A
$V_{OC}$	Open-circuit voltage	50.8	V
$I_{SC}$	Short-circuit current	8.86	A
$K_{V}$	Temp. coefficient of $V_{OC}$	−0.3551	%/$^\circ$C
$K_{I}$	Temp. coefficient of $I_{SC}$	0.02399	%/$^\circ$C
$R_{S}$	Series resistance of PV cell	0.41296	$\Omega$
$R_{P}$	Parallel resistance of PV cell	270.25	$\Omega$
$N_{cell}$	Cells per module	80	–
$N_{sm}$	Number of series modules	6	–

**Fig. 1:**
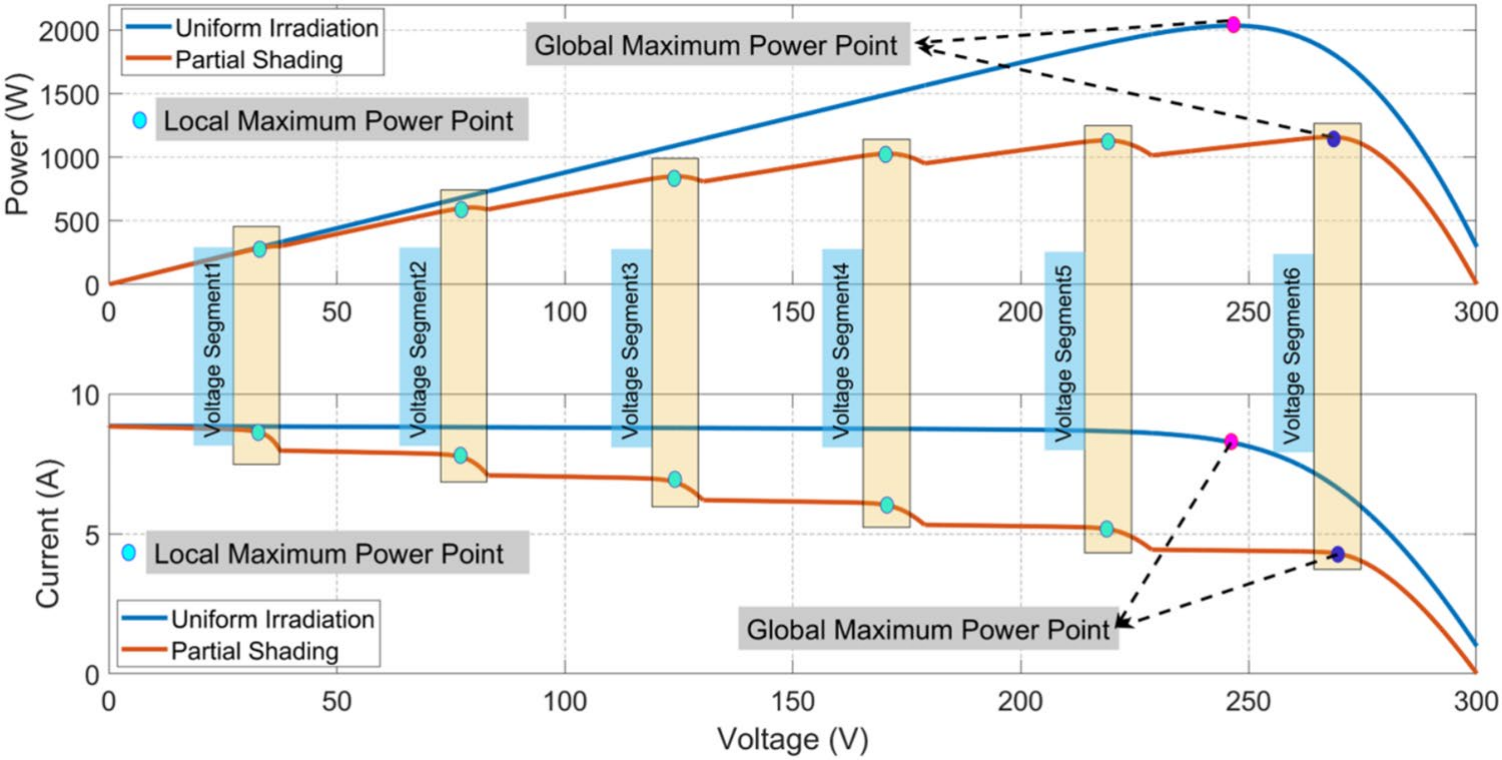
*P*–*V* and *I*–*V* curves of the PV array under uniform irradiance and PSC.

When all series-connected PV modules are subjected to the same irradiance level, the array operates under uniform irradiance conditions, and only a single GMPP exists. Although the GMPP location varies with temperature and irradiance, conventional MPPT algorithms can track the maximum power reliably when the power–voltage characteristic exhibits a single peak. In contrast, under nonuniform irradiance (partial shading), multiple power maxima emerge, as illustrated in Fig. 1. In this case, only one of these points corresponds to the GMPP, whereas the remaining peaks constitute LMPPs, and the MPPT algorithm must identify the true GMPP. For a PV string, it is possible for the number of distinct MPPs to be as high as the number of series-connected modules. Depending on the irradiance distribution, the number of LMPPs may therefore vary, with an upper bound equal to the number of series-connected modules.

Since six modules are connected in series in the simulation study, Fig. 1 indicates that LMPPs may arise across six distinct voltage regions. MPPT algorithms are particularly prone to misidentifying the GMPP when similar power levels occur in different voltage regions, and when the GMPP lies in low-voltage regions. Therefore, an MPPT algorithm can be considered truly effective only if it maintains high tracking efficiency under all such challenging operating conditions.

## M-DHO algorithm

The dhole (*Cuon alpinus*), also known as the Asiatic wild dog, inhabits forests and grasslands across Central, East, and Southeast Asia. Dholes are notable for their complex social organization, cooperative hunting behavior, and advanced vocal communication. Inspired by these natural traits, the DHO algorithm is a recently proposed metaheuristic designed to solve complex optimization problems. As summarized in [49], the main behavioral principles underlying DHO can be outlined as follows:

- **Lead vocalizer and social structure:** Dholes live in packs ranging from small family units to large groups (up to several dozen individuals). Each pack may be guided by a lead vocalizer that directs collective movement and coordinates hunting efforts through vocal signals.
- **Vocal communication and adaptive control:** During hunting, vocal signals convey information about prey location and guide group motion. In DHO, this communication mechanism is abstracted into an adaptive control strategy that regulates the search dynamics.
- **Cooperative hunting and solution adjustment:** Pack members employ diverse hunting tactics to capture prey. The pack disperses to select a target, while some individuals steer the prey toward preferred directions. Analogously, DHO updates candidate solutions by adjusting their positions in real time with respect to the current best solutions and the distribution of the population.
- **Territorial instincts and boundary constraints:** In addition to cooperation, dholes maintain hunting territories, preventing prey from escaping beyond a defined region and thereby reducing risk. In DHO, this behavior is represented through boundary-handling mechanisms that keep candidate solutions within feasible search limits.

The efficiency of optimization algorithms is commonly evaluated based on the speed at which they converge to an optimal or near-optimal solution. Although rapid convergence can be advantageous, it may also increase the risk of premature convergence to suboptimal solutions, particularly in complex search spaces containing numerous local optima. Therefore, an effective optimization process requires a balanced trade-off between exploration, which involves searching new regions of the solution space, and exploitation, which focuses on refining already promising solutions. The DHO algorithm builds upon these principles by integrating hierarchical leadership dynamics with cooperative hunting strategies, thereby distinguishing itself from conventional swarm-based and population-based optimization approaches. Its voice-based routing mechanism introduces a novel framework for managing the exploration–exploitation balance, contributing to the development of adaptive and self-organizing optimization techniques. Especially with Levy flight support, the DHO algorithm eliminates the problems of fast convergence and getting stuck in local minima. When the randomly generated number is below 0.5 during the Exploration phase of the algorithm, the randomly generated number becomes equal to the fitness function value. This reduces errors caused by rapid convergence. In the proposed algorithm, by applying the Levy Flight algorithm in the specified situation, both the rapid convergence problem is eliminated and the global maximum point is reached with less error through a systematic approach. In the DHO algorithm, vocal communication is modeled as in Eq. 6. As the iterations proceed, the influence of the lead vocalizer gradually decreases, thereby facilitating a transition from exploration to exploitation.

6 $$\begin{aligned} V = 2 - t\times \left( \frac{2}{Max_{iter}}\right) \end{aligned}$$

Here, *V* denotes the vocalization effect, *t* is the current iteration index, and $Max_{iter}$ is the maximum number of iterations. The exploration–exploitation decision determines whether a search agent explores new regions or exploits the best-known solutions. To control the movement intensity of search agents, a scaling factor *B* is defined as in Eq. 7.

7 $$\begin{aligned} B = V\times r \end{aligned}$$

where *r* is a random number uniformly sampled from [0, 1]. To induce controlled oscillations in the movement dynamics, the coefficient *C* is computed using a sinusoidal function as in Eq. 8.

8 $$\begin{aligned} C = r + \sin \left( r\times \pi \right) \end{aligned}$$

This sinusoidal component promotes controlled variations in the search trajectory, which reduces the probability of search agents being trapped in local optima while preventing premature convergence. In the next step, the adjusted Euclidean distance between each search agent and the lead vocalizer is computed as in Eq. 9.

9 $$\begin{aligned} \overrightarrow{D}_{lead} = \left| C\times \overrightarrow{X}_{LV}^{\,2} - \overrightarrow{D}_{i}^{\,2}\right| \end{aligned}$$

where $\overrightarrow{X}_{LV}$ denotes the lead vocalizer position and $\overrightarrow{D}_{i}$ represents the current position of the *i*th search agent. The coefficient *C* yields a more aggressive search behavior during the early iterations and gradually promotes a more refined search as the iterations progress. In the next stage, the new position of the search agent is updated as in Eq. 10.

10 $$\begin{aligned} \overrightarrow{X}_{lead} = \overrightarrow{X}_{LV}\times B\times \sqrt{\overrightarrow{D}_{lead}} \end{aligned}$$

This update rule enables each agent to move toward the lead vocalizer with a step size scaled by $\sqrt{\overrightarrow{D}_{lead}}$, which smooths the movement dynamics and helps mitigate irregular jumps. To keep search agents within admissible bounds and prevent excessive

11 $$\begin{aligned} \overrightarrow{D}_{i} = {\left\{ \begin{array}{ll} \textrm{lb} + \left( \textrm{ub}-\textrm{lb}\right) \times \textrm{rand}(\textrm{dim}), & \overrightarrow{D}_{i} < \textrm{lb} \;\text {or}\; \overrightarrow{D}_{i}> \textrm{ub},\\ \overrightarrow{D}_{i}, & \text {otherwise}, \end{array}\right. } \end{aligned}$$

where $\textrm{lb}$ and $\textrm{ub}$ denote the lower and upper bounds of the search space, respectively, and $\textrm{rand}(\textrm{dim})$ generates a random vector that relocates any infeasible solution back into the feasible domain. At the end of each iteration, the agent with the best fitness value is assigned as the lead vocalizer, as expressed in Eq. 12.

12 $$\begin{aligned} \overrightarrow{X}_{LV} = {\left\{ \begin{array}{ll} \overrightarrow{D}_{i}, & \text {if } f\left( \overrightarrow{D}_{i}\right) < f\left( \overrightarrow{X}_{LV}\right) ,\\ \overrightarrow{X}_{LV}, & \text {otherwise}, \end{array}\right. } \end{aligned}$$

Single-objective optimization algorithms encompass both deterministic and stochastic approaches. Among these, stochastic methods are generally more effective in addressing problems characterized by multiple local maxima. Consequently, optimization algorithms require the continuous development of adaptive mechanisms that balance exploration and exploitation. In the DHO algorithm, the Leader Vocalist’s position in the search space functions as a focal point that guides other candidate solutions toward promising regions. The algorithm adaptively balances global exploration and local exploitation by dynamically adjusting the influence of the Leader Vocalist. This mechanism ensures that the search process remains diverse and avoids premature convergence to suboptimal solutions. Such a hierarchical structure distinguishes DHO from other swarm-based algorithms by introducing a flexible leadership mechanism that adapts according to the progress of the search. The DHO algorithm employs a multi-stage leadership model in which leadership dynamically shifts among agents based on real-time performance metrics. This transition facilitates adaptive decision-making, enhances information diffusion across the population, and prevents stuck in local optima by encouraging diverse exploration.In the DHO algorithm, during the exploration phase, if the randomly generated number is less than 0.5, the duty cycle used to switch the DC–DC converter is set equal to this randomly generated value. This mechanism helps prevent the algorithm from becoming trapped in local minima during exploration. However, it may also cause the search process to move away from the global maximum. In the proposed algorithm, when the randomly generated number in the exploration phase is less than 0.5, the Levy Flight strategy is employed. This modification enhances the algorithm’s ability to escape local maxima while simultaneously improving its capability to converge toward the global optimum. The fitness function evaluates the quality of each candidate solution; if a search agent achieves a better fitness value than the current lead vocalizer, it replaces the lead vocalizer in the subsequent iteration. The algorithm of the proposed M-DHO algorithm is presented in Algorithm 1. Accordingly, in each iteration of DHO, *V* is computed and the algorithm switches between the exploration and exploitation phases. In the exploration phase, if a randomly generated number is less than 0.5, the Levy flight operator is activated and the newly generated value is assigned to $\overrightarrow{D}_{lead}$. Thus, for each instance in which the random number is below 0.5, $\overrightarrow{D}_{lead}$ is recomputed via the Levy flight relations to promote convergence toward a more suitable solution. As shown in Eq. 13, a new candidate solution (i.e., a new duty cycle) is generated using the Levy flight update^56^:

**Algorithm 1:**
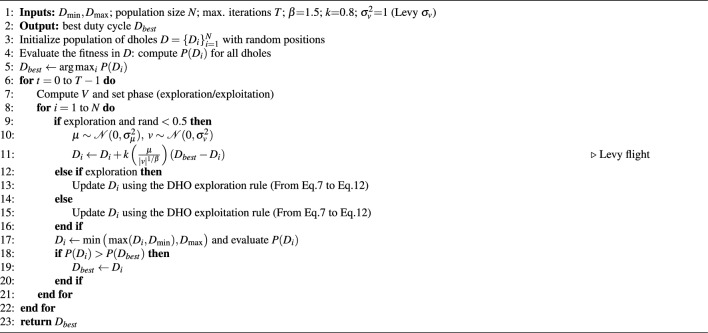
Pseudocode of the proposed M-DHO–based MPPT method.

13 $$\begin{aligned} \overrightarrow{D}_{i}^{t+1} = \overrightarrow{D}_{i}^{t} + \alpha \odot \textrm{Levy}(\lambda ), \quad i = 1,2,\ldots ,n \end{aligned}$$

Here, *n* is the problem dimension, $\odot$ denotes the Hadamard (element-wise) product, and $\textrm{Levy}(\lambda )$ represents a random step vector drawn from a Levy distribution. The step-size factor $\alpha$ can be written as in Eq. 14.

14 $$\begin{aligned} \alpha = \alpha _{0}\left( \overrightarrow{D}_{best} - \overrightarrow{D}_{i}\right) \end{aligned}$$

The Levy flight term can be simplified as in Eq. 15.

15 $$\begin{aligned} \alpha _{0}\left( \overrightarrow{D}_{best} - \overrightarrow{D}_{i}\right) \odot \textrm{Levy}(\lambda ) \approx k\left( \frac{\mu }{\left| v\right| ^{1/\beta }}\right) \left( \overrightarrow{D}_{best} - \overrightarrow{D}_{i}\right) \end{aligned}$$

where $\beta = 1.5$, *k* is a user-defined scaling constant, and $\mu$ and *v* are random variables drawn from normal distributions, as given in Eq. 16.

16 $$\begin{aligned} \mu \sim \mathcal {N}\left( 0,\sigma _{\mu }^{2}\right) , \quad v \sim \mathcal {N}\left( 0,\sigma _{v}^{2}\right) \end{aligned}$$

with $\sigma _\mu$ and $\sigma _v$ defined as in Eq. 17.

17 $$\begin{aligned} \sigma _{\mu } = \left( \frac{\Gamma \left( 1+\beta \right) \sin \left( \pi \beta /2\right) }{\Gamma \left( (1+\beta )/2\right) \beta \,2^{(\beta -1)/2}}\right) ^{1/\beta }, \quad \sigma _{v} = 1. \end{aligned}$$

Otherwise (i.e., for random numbers greater than 0.5), $\overrightarrow{D}_{lead}$ is computed based on the update rules, and the new position of the search agent is determined.

## Simulation and results

The simulations were conducted in the MATLAB/Simulink environment using six series-connected Kyocera Solar KU340-8BCA PV modules. Under standard test conditions, the PV system can deliver a maximum power of $2042\,\textrm{W}$ at an output voltage of $247.2\,\textrm{V}$. A DC–DC converter was employed at the PV output to run the MPPT algorithm. A $100\,\Omega$ resistive load was connected at the converter output. The MPPT algorithm directly controlled the converter switch by generating the duty cycle, and a PWM signal with a switching frequency of $20\,\textrm{kHz}$ was used for gating.

A boost type DC-DC converter is used at the output of the PV system. The duty cycle generated by the MPPT algorithm switches the DC-DC converter. To ensure maximum power is transferred to the load at the output of the DC-DC converter, the MPPT algorithm sets the power of the PV system to its maximum. The sampling time of the simulation is taken 1 $\mu s$. The current and voltage sampling of the PV system is determined as 10 $\mu s$. When the *Q* switch in the boost converter is on, the inductance voltage can be written as shown in Eq. 18. The duration that the switch *Q* stays in transmission can be written as $\Delta t$, *D*.*Ts*. Here, *D* denotes the duty cycle of the switch *Q*, and $T_s$ denotes the total time that the switch stays in the transmission and cut off, that is, the switching period. The duration in which the switch *Q* is cut-off can be written as $(1-D)T_s$. In Eq. 19, the change in the current in the L inductance in the cut-off state of the *Q* switch is seen.

18 $$\begin{aligned} & V_s(t)=L\frac{dI_L}{dt}\qquad \text {and}\qquad \frac{\Delta I_L}{\Delta t}=\frac{V_s}{L} \end{aligned}$$

19 $$\begin{aligned} & \Delta I_L=\frac{V_s-V_0}{L}(1-D)T_s \end{aligned}$$

The sum of the inductor current variation in the ON and OFF states of the switch is zero, as given in Eq. 20.

20 $$\begin{aligned} \frac{V_s}{L}DT_s+\frac{V_s-V_0}{L}(1-D)T_s=0 \end{aligned}$$

Using Eq. 20, the relationship between the boost-converter input and output voltages is obtained as in Eq. 21.

21 $$\begin{aligned} \frac{V_0}{V_s}=\frac{1}{1-D} \end{aligned}$$

The minimum inductance required for continuous-conduction-mode operation is given in Eq. 22^57^.

22 $$\begin{aligned} L_{\min }=\frac{(1-D)^2DR}{2f_s} \end{aligned}$$

To keep the output-voltage ripple within the desired limits, the minimum capacitance is calculated as given in Eq. 23

23 $$\begin{aligned} C_{\min }=\frac{D}{R V_r f_s} \end{aligned}$$

The boost-converter parameters are presented in Table 2.

**Table 2:** Parameters of boost converter.

Parameter	Value	Unit
*L*	8.7	$\mu$H
*C*	59	$\mu$F
*R*	100	$\Omega$
$f_s$	20	kHz

**Fig. 2:**
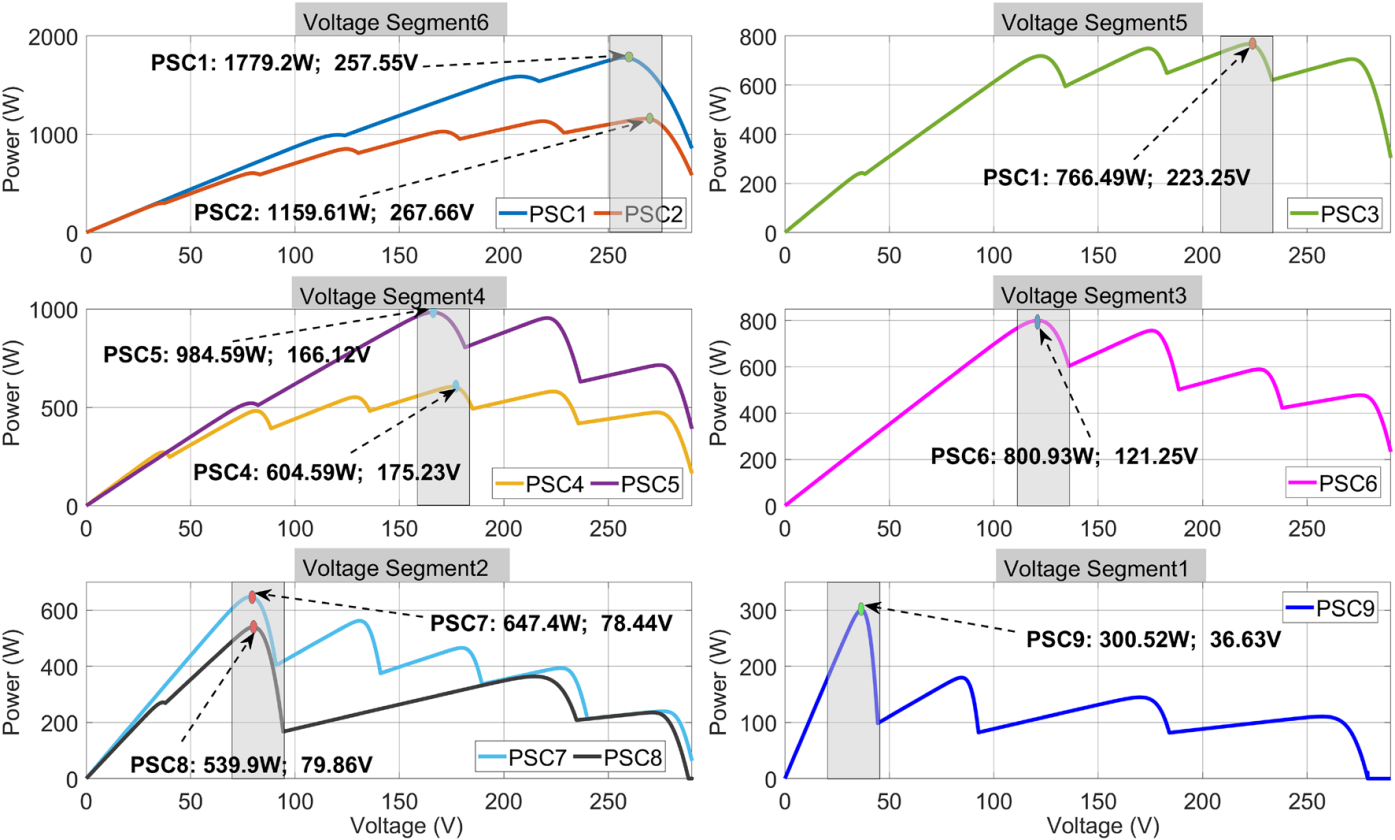
*P*–*V* characteristics of the PV array under the nine partial shading scenarios.

The partial shading scenarios (see Appendix Fig. 6) were defined in the simulations. In these scenarios, PSC1 corresponds to the highest irradiance level, whereas PSC6 represents the lowest irradiance condition. In PSC1–PSC6 and PSC8, three series-connected modules share the same irradiance level; this pattern differs in the remaining scenarios. Consequently, different numbers of LMPPs were created across distinct voltage regions to assess the effectiveness of the proposed method.

**Fig. 3:**
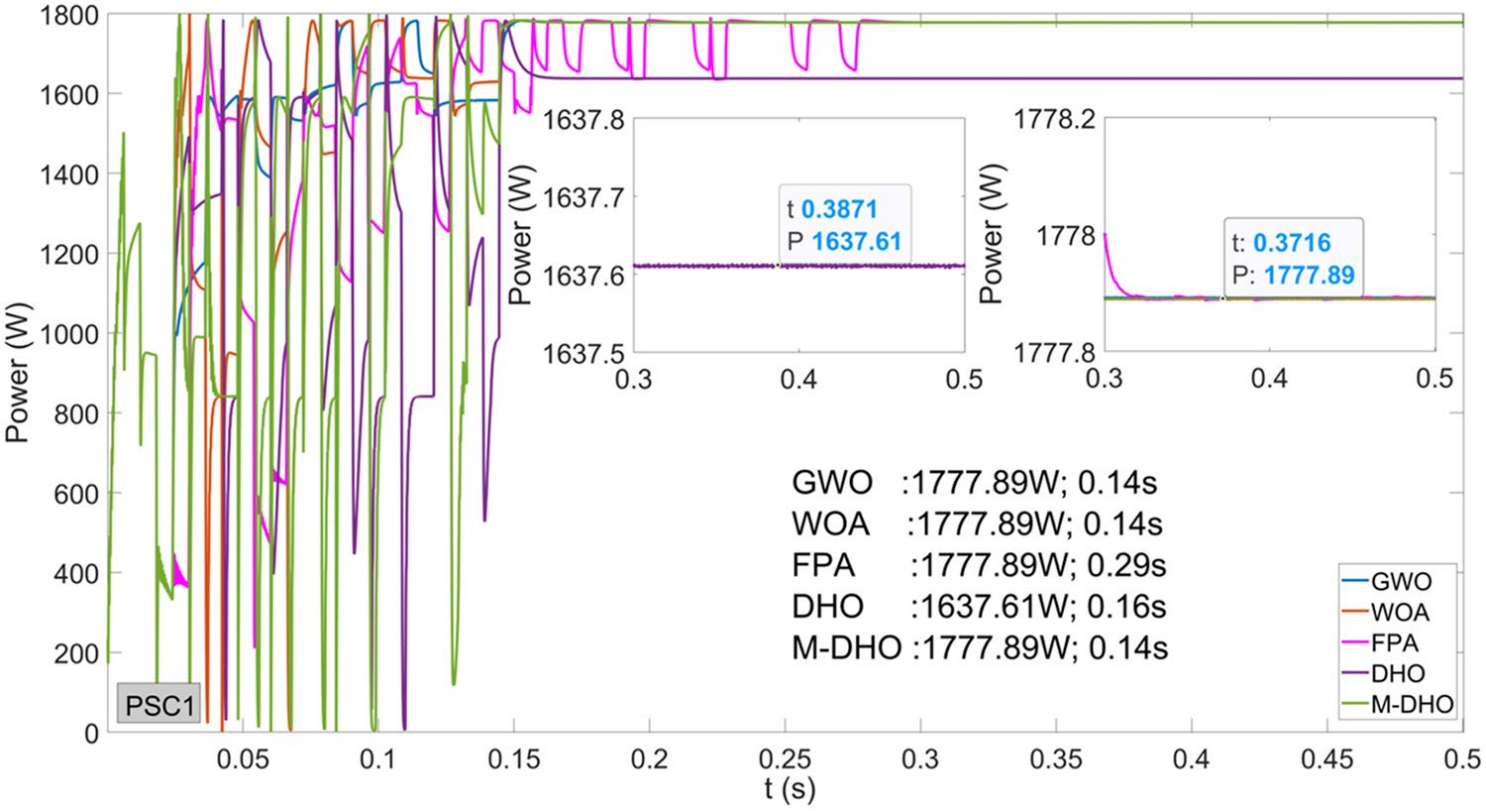
Power waveforms under the partial shading scenario (PSC1). Other scenarios are presented in Appendix 8.

Figure 2 illustrates the maximum power values obtained in six distinct voltage regions. Accordingly, PSC1 and PSC2 are located in the sixth voltage region, and the ability of the proposed method to identify high- and low-magnitude maxima within the same voltage region is investigated. In the PSC3 and PSC4 scenarios constructed in the fifth and sixth voltage regions, the maximum power points in three adjacent voltage regions are very close to each other. The capability of the proposed algorithm to locate the GMPP among these closely spaced peaks is therefore examined under these particularly challenging conditions. Moreover, PSC7–PSC8 and PSC9 correspond to cases in which the maximum power occurs in the low-voltage region. MPPT algorithms often face difficulties in such low-voltage regions because the duty-cycle search interval becomes wide when scanning between the highest- and lowest-voltage regions; searching over a wide interval is more challenging and typically increases the tracking time. All simulation studies were carried out with identical initial conditions and iterations increasing at equal time intervals. The initial duty-cycle values were selected as 0.2, 0.4, 0.6, and 0.8. The simulations were repeated for the GWO, WOA, FPA, DHO, and M-DHO algorithms. Important parameters of the optimization algorithms are given in Table 3.

**Table 3:** Parameters of optimization algorithms.

Optimization Method	Parameters	Value
GWO	Max. iteration	20
	Search agents	4
	Encircling coeff.	$[0\;2]$
	$\vec {a}$	Linearly decreased from 2 to 0
FPA	Pollen Number	4
	Probability (*P*)	0.8
	Levy exponent ($\lambda$)	1.5
WOA	Max. iteration	20
	Search agents	4
	*r*	Random $[0\;1]$
	$\vec {a}$	Linearly decreased from 2 to 0
M-DHO	Max. iteration	20
	Search agents	4
	*V*	Linearly decreased from 2 to 0
	$\vec {a}$	Linearly decreased from 2 to 0
	Levy exponent ($\lambda$)	1.5

For PSC1, the resulting output powers are presented in Fig. 3. Except for the DHO algorithm, the compared MPPT methods operated with an efficiency of 99.92% by converging very close to the maximum power. The FPA reached the maximum power latest, with a convergence time of 0.29 s. The proposed method reached the maximum power in 0.14 s, thereby demonstrating both high speed and high efficiency. The output powers obtained under PSC2 are shown in Fig. 8b. Under this condition, the FPA did not reach the exact maximum power within 0.5 s, confirming its relatively slow response in the first two scenarios. By contrast, the WOA, DHO, and M-DHO algorithms converged very close to the maximum power and continued operating with an efficiency of 99.99% after 0.15 s. In the voltage region containing PSC1 and PSC2, the proposed method achieved superior speed and efficiency. In addition, the WOA also exhibited high performance in this voltage region. PSC3 and PSC4 represent challenging atmospheric scenarios for MPPT algorithms because they yield very similar power values in different voltage regions; consequently, algorithm performance in these regions can significantly affect overall tracking efficiency. The output powers obtained under PSC3 are shown in Fig. 8c. The proposed M-DHO algorithm clearly outperformed the other methods, reaching the maximum power in 0.16 s and operating at a steady-state tracking efficiency of 99.54%. While M-DHO achieved 762.99 W, the closest competing results were obtained by GWO, WOA, and FPA, each producing 716.64 W. As shown in Fig. 8d, under PSC4, all methods except GWO produced 599.96 W, indicating nearly identical performance. However, consistent with the other scenarios, the FPA remained the slowest method, reaching the maximum power at 0.34 s. PSC5 exhibits a *V*–*P* characteristic in which MPPT algorithms have a high probability of being trapped at an LMPP. As observed in Fig. 8e, under PSC5, the GWO, FPA, and M-DHO algorithms approached the maximum power by producing 984.33 W. Nevertheless, the FPA again showed the slowest convergence, reaching the maximum at 0.30 s. In contrast, WOA and DHO exhibited the lowest efficiencies, producing 947.73 W. The output powers obtained under PSC6 are shown in Fig. 8f. In this scenario, the proposed method achieved its lowest efficiency, producing 762.04 W with a tracking efficiency of 98.89%. The GWO and FPA attained the highest efficiencies (99.98%), whereas the power produced by M-DHO remained at an acceptably high level. PSC7, PSC8, and PSC9 correspond to scenarios in which the maximum power occurs in low-voltage regions. Figure 8g and h present the output powers obtained under PSC7 and PSC8, respectively, where GWO and M-DHO achieved the highest efficiencies. Under PSC9 (Fig. 8i), WOA and M-DHO exhibited the best performance. Across all low-voltage scenarios (PSC7–PSC9), the proposed M-DHO algorithm maintained high tracking efficiency, whereas the efficiencies of the other algorithms varied. Moreover, the high convergence speed of M-DHO was also observed in these scenarios. Simulation results indicate that the MPP occurs within six distinct voltage regions. In several scenarios, the power levels in these regions are very close to one another, which increases the difficulty of accurately identifying the global maximum. The wide scanning range required during the search process, combined with the small differences between local power levels, presents a significant challenge for MPPT algorithms. Scenarios PSC2, PSC3, PSC4, and PSC5 particularly exhibit these characteristics. In these challenging scenarios, MPPT algorithms except for the FPA algorithm terminated within the same number of iterations. However, under complex operating conditions, algorithms may converge to different voltage regions despite completing the same number of iterations, indicating that they may become trapped in local optima. Therefore, achieving the maximum power point with fewer iterations is an important performance metric that reflects the tracking speed and effectiveness of the algorithm. As a result of the same number of iterations, the M-DHO algorithm was able to achieve operation in the maximum power region in the same iteration in all scenarios.

**Table 4:** Comparison of MPPT algorithms under nine partial shading scenarios.

Scenario	GMPP (W)	Power (W)					Tracking Efficiency (%)					Tracking Speed (s)				
		GWO	WOA	FPA	DHO	M-DHO	GWO	WOA	FPA	DHO	M-DHO	GWO	WOA	FPA	DHO	M-DHO
PSC1	1779.2	1777.89	1777.89	1777.89	1637.61	1777.89	99.92	99.92	99.92	92.04	99.92	0.14	0.14	0.29	0.16	0.14
PSC2	1159.61	1061.56	1159.54	1104.36	1159.54	1159.54	91.54	99.99	95.23	99.99	99.99	0.15	0.15	0.50	0.15	0.15
PSC3	766.49	716.64	716.64	716.64	712.23	762.99	93.49	93.49	93.49	92.92	99.54	0.15	0.15	0.28	0.15	0.16
PSC4	604.59	537.09	599.96	599.96	599.96	599.96	88.83	99.23	99.23	99.23	99.23	0.15	0.16	0.34	0.16	0.16
PSC5	984.59	984.33	947.73	984.33	947.73	984.33	99.97	96.25	99.97	96.25	99.97	0.14	0.16	0.30	0.16	0.15
PSC6	800.93	800.81	792.04	800.81	797.04	792.04	99.98	98.89	99.98	99.51	98.89	0.14	0.14	0.39	0.14	0.15
PSC7	647.4	644.26	588.07	588.07	644.26	644.26	99.51	90.83	90.83	99.51	99.51	0.14	0.16	0.35	0.15	0.15
PSC8	539.9	536.42	536.42	536.42	536.42	536.42	99.35	99.35	99.35	99.35	99.35	0.13	0.16	0.39	0.16	0.16
PSC9	300.52	196.99	289.77	227.66	277.27	289.77	65.54	96.42	75.75	92.26	96.42	0.17	0.17	0.50	0.15	0.17
Mean	842.58	806.22	823.12	815.12	812.45	838.58	95.68	97.69	96.74	96.42	99.52	0.15	0.15	0.37	0.15	0.15

**Fig. 4:**
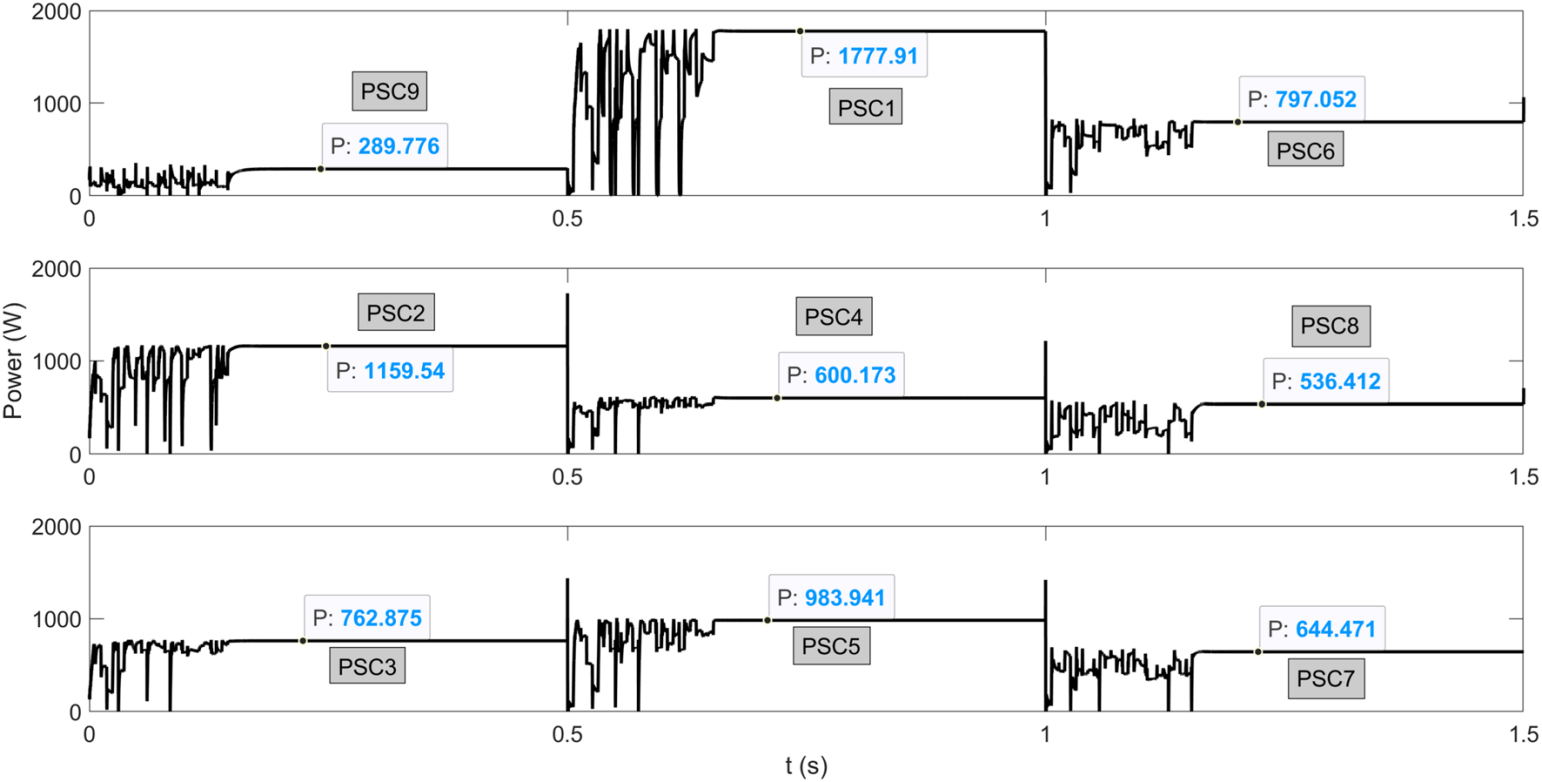
Output power obtained by the proposed M-DHO algorithm under sequentially applied atmospheric scenarios.

Considering all scenarios in detail, M-DHO exhibited superior performance under all atmospheric conditions except PSC6. Even in PSC6, the power obtained by the proposed method remained very close to the maximum power point and achieved an acceptable efficiency. When the results of all nine scenarios are averaged, the proposed M-DHO method attains the highest mean tracking efficiency of 99.52%, with a mean convergence time of 0.15 s. Table 4 summarizes the extracted power, tracking efficiency, and convergence time for all benchmark algorithms across the nine scenarios.

Figure 4 presents the output power produced when the scenarios are applied sequentially. In all three sequential test cases, the proposed algorithm successfully tracked the maximum power. By applying PSC9–PSC1–PSC6 sequentially, the algorithm performance was demonstrated during transitions from the low-voltage region to the high-voltage region and then from the high-voltage region to a medium-voltage region. This confirms that the proposed method can operate reliably and with high efficiency across substantially different voltage regions and power levels. When PSC4 and PSC8 were applied consecutively, the algorithm maintained high performance despite the sequential application of two challenging scenarios. Since PSC4 and PSC8 yield similar power levels, the algorithm was also tested under atmospheric changes with closely spaced power values. Finally, PSC3 and PSC5, which produce their maximum power points in nearby voltage regions, were applied to the PV system, and the algorithm was again observed to extract power with high efficiency.

Appendix Fig. 7 shows the PV array voltage values obtained using the proposed M-DHO method. Under all atmospheric conditions, the operating voltages were produced within the expected voltage regions. Accordingly, the proposed algorithm can reach the maximum-power regions even under challenging conditions.

These results indicate that the M-DHO algorithm remains effective under rapidly changing atmospheric conditions. Given the computed mean convergence time of 0.15 s, the proposed method is expected to capture the maximum power even when the atmospheric conditions change at approximately 0.2 s intervals.

**Fig. 5:**
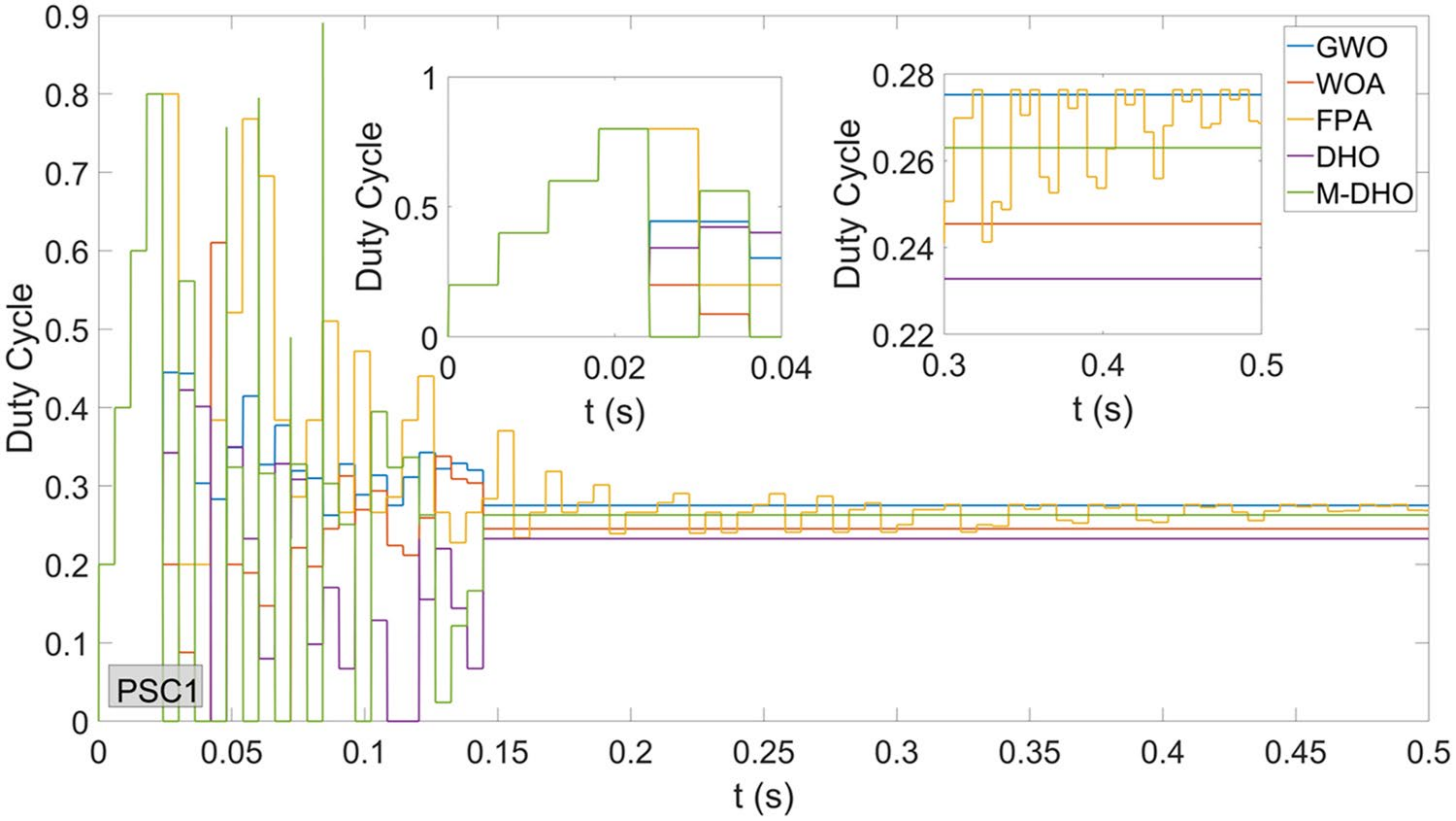
Duty-cycles for PSC1. Other scenarios are presented in Appendix 9.

**Fig. 9:**
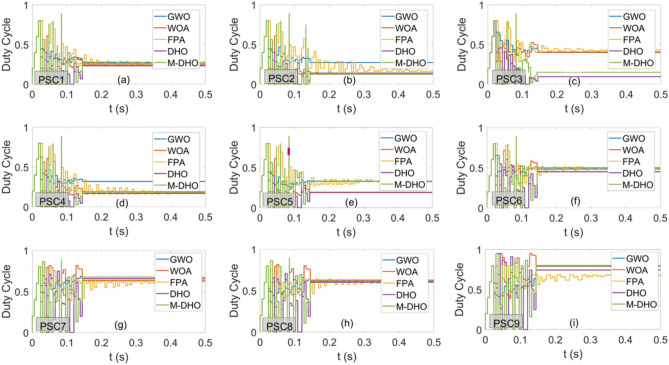
Duty cycles under partial shading scenarios PSC1–PSC9.

As illustrated in Fig. 5, the duty cycle values generated by the MPPT algorithms across nine different scenarios vary approximately between 0.1 and 0.8 under steady-state conditions. In many studies reported in the literature, duty cycle variations are typically examined within a narrower operating range. However, conducting the search over a wider duty cycle interval introduces greater complexity and provides a more rigorous evaluation of algorithm performance. The proposed algorithm successfully identified the voltage region corresponding to the maximum power point within this wide duty cycle range. In Fig. 9a (PSC1), all algorithms managed to generate power close to the maximum power as expected. Although the DHO algorithm generated slightly lower power in this region, it correctly found the voltage region. While preventing early convergence, it moved away from the maximum power value. Fig. 9b presents the duty cycles obtained under PSC2 conditions. In this case, the GWO algorithm generated a different duty cycle and became trapped in a local optimum corresponding to another voltage region. This behavior is mainly due to the presence of power levels close to the global maximum in adjacent voltage regions, which makes PSC2 a challenging scenario. The FPA algorithm was unable to reach the maximum power within 0.5 s. However, since it correctly identified the appropriate voltage region, it could potentially have reached the maximum power if the simulation had been extended. The simulation duration was limited because the other algorithms reached the maximum power within a shorter time. As shown in Fig. 9c (PSC3), the DHO and M-DHO algorithms generated duty cycles different from those of the other algorithms, which converged to other voltage regions. PSC3 represents a particularly challenging scenario in which four adjacent voltage regions produce very similar power levels. In this case, the M-DHO algorithm successfully reached the correct voltage region by effectively balancing exploration and exploitation, and the incorporation of the Levy Flight mechanism enabled it to achieve the maximum power point. Although the DHO algorithm moved toward the correct voltage region, it ultimately deviated from the maximum power point. As seen in Fig. 9d (PSC4), the other algorithms, except GWO, showed superior performance. Although PSC4 is a challenging scenario, the voltage region it operated in was an easily achievable result for the algorithms. As shown in Fig. 9e (PSC5), the DHO and WOA algorithms generated different duty cycles and consequently operated in different voltage regions, resulting in lower power output. In contrast, the remaining algorithms successfully converged toward the optimal region. Fig. 9f illustrates the PSC6 scenario, which represents a relatively less complex condition. Accordingly, all algorithms generated duty cycles that were close to one another and converged to the correct voltage region. In this case, the M-DHO algorithm produced slightly lower power compared to the others.As seen in Fig. 9g and h (PSC7-PSC8), the algorithms produced duty cycles close to each other. All algorithms reached the maximum power region. As seen in Fig. 9i, the proposed algorithm and the WOA algorithm show a significant power generation advantage. In this region, small differences in duty cycles cause significant changes in power values.

## Conclusions

MPPT algorithms are expected to capture the maximum power point rapidly and to maintain high tracking efficiency under challenging atmospheric conditions. In this study, a PV system was modeled in the MATLAB/Simulink environment using six series-connected Kyocera Solar KU340-8BCA modules, producing a maximum power of 2042 W under standard test conditions. Nine distinct PSCs were created across six voltage regions, and the performance of GWO, WOA, FPA, DHO, and the proposed M-DHO algorithms was evaluated. Among the core strengths of the DIO algorithm are its adaptive parameters that dynamically balance exploration and exploitation, its effective avoidance of local optima through coordinated search agent movements, and its robustness across a variety of optimization environments. These features contribute to the algorithm’s high convergence speed and accuracy, making it a versatile tool for a wide range of optimization problems. One major challenge is the lack of a formal theoretical convergence proof, which leaves it an open area of research in nature-inspired optimization.

The proposed M-DHO algorithm is a novel optimization approach obtained by integrating the DHO algorithm with a Levy flight strategy. The designed PSC set includes particularly challenging cases, such as (i) closely spaced power peaks occurring in different voltage regions and (ii) scenarios in which the global maximum power point lies in a low-voltage region, thereby enabling a rigorous assessment of tracking robustness.

Simulation results demonstrate that the proposed method converges to the maximum power point with high speed and high efficiency under all PSC scenarios except PSC6, where the tracking efficiency remains at an acceptably high level. When averaged over all scenarios, M-DHO achieved an average extracted power of 838.58 W and an average tracking efficiency of 99.52%, outperforming GWO (95.68%), WOA (97.69%), FPA (96.74%), and DHO (96.42%). Moreover, M-DHO reached the maximum power with a mean convergence time of 0.15 s, whereas FPA exhibited the slowest performance with an average tracking time of 0.37 s. The proposed algorithm has been implemented in a simulation environment. In real-world applications, high-level controllers are needed due to the high complexity and computational intensity. The implementation difficulty of the proposed algorithm is moderate. Therefore, it can be implemented with currently used inexpensive microcontrollers. On the other hand, current and voltage measurements should be purified from noise using appropriate filters.

Overall, the results confirm that the proposed M-DHO-based MPPT algorithm can operate with high efficiency and high speed under demanding PSC conditions. As with many metaheuristic algorithms, one of the fundamental challenges in the DHO algorithm is the lack of formal theoretical convergence proof in nature-inspired optimization. Unlike deterministic gradient-based methods with well-defined convergence properties, metaheuristic algorithms rely on stochastic exploration and exploitation, which makes theoretical analysis more complex. Another challenge is parameter sensitivity, as DHO’s performance depends on parameter selection. Developing self-adaptive tuning mechanisms can improve performance. Furthermore, using hybrid algorithms can optimize the balance between exploration and exploitation. Future work will focus on developing a hybrid MPPT technique by combining M-DHO with other optimization strategies to reduce the number of iterations required to reach the maximum power point.

## Figures and Tables

**Fig. 6:**
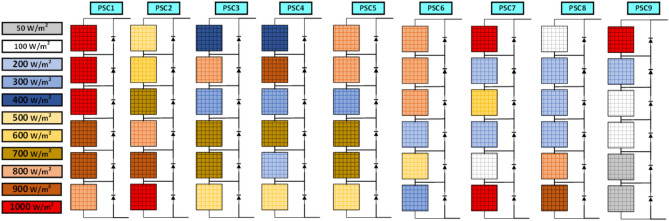
Module irradiance patterns for the nine partial shading scenarios.

**Fig. 7:**
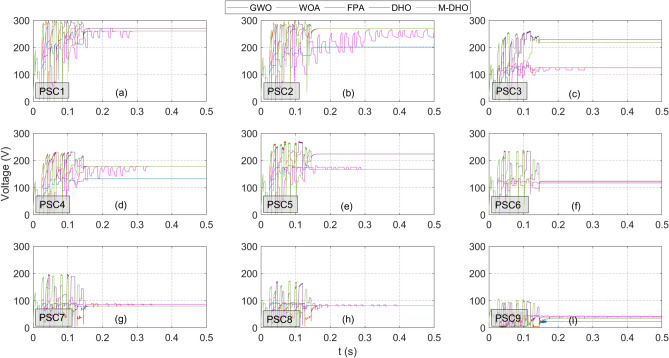
PV system voltages under partial shading scenarios.

**Fig. 8:**
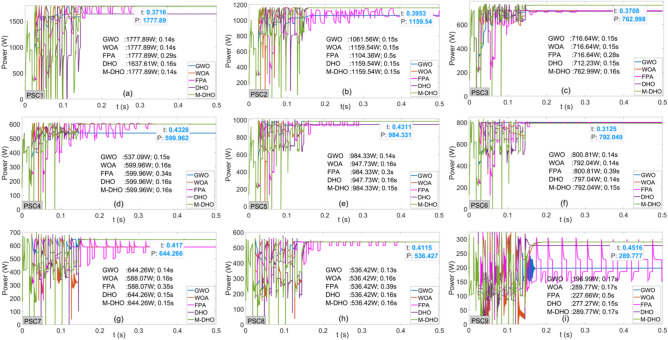
Power waveforms under partial shading scenarios PSC1–PSC9.

## Data Availability

The data that support the findings of this study are publicly available at https://github.com/rcelikel/Modified-Dhole-Inspired-Optimization-based-MPPT. Additional information is available from the corresponding author upon reasonable request.

## A Appendix

### A.1 Partial shading scenarios

See Fig. 6.

### A.2 PV system voltages

See Fig. 7.

### A.3 Power waveforms under PSC

See Fig. 8.

### A.4 Duty cycles under PSC

See Fig. 9.

## Abbreviations

CSA: Cuckoo Search Algorithm
DHO: Dhole-Inspired Optimization
FPA: Flower Pollination Algorithm
GMPP: Global Maximum Power Point
GWO: Grey Wolf Optimization
InC: Incremental Conductance
LMPP: Local Maximum Power Point
M-DHO: Modified Dhole-Inspired Optimization
MPPT: Maximum Power Point Tracking
PSC: Partial Shading Condition
PSO: Particle Swarm Optimization
PV: Photovoltaic
WOA: Whale Optimization Algorithm
